# Digital Twin–Based Virtual Hospital Platform for IT Outage Disaster Response Training: Implementation and Evaluation Study

**DOI:** 10.2196/93135

**Published:** 2026-08-21

**Authors:** SungA Bae, Ye Ji Kim, Min Woo Lee, Soo Jeong Kim, Jin Young Park

**Affiliations:** 1 Center for Digital Health Yongin Severance Hospital Yonsei University Health System Yongin, Seoul Republic of Korea; 2 Department of Cardiology Yongin Severance Hospital, Yonsei University College of Medicine Yongin, Gyeonggi-do Republic of Korea; 3 Division of Planning and Management Office of Medical Information Technology Yongin Severance Hospital, Yonsei University College of Medicine Yongin, Gyeonggi-do Republic of Korea; 4 Department of Hematology & Oncology Yongin Severance Hospital, Yonsei University College of Medicine Yongin, Gyeonggi-do Republic of Korea; 5 Department of Psychiatry Yongin Severance Hospital, Yonsei University College of Medicine Yongin, Gyeonggi-do Republic of Korea; 6 Institute of Behavioural Science in Medicine Yongin Severance Hospital, Yonsei University College of Medicine Yongin, Gyeonggi-do Republic of Korea

**Keywords:** digital twin, virtual hospital simulation, IT outage, disaster preparedness, health information systems, simulation training, survey feedback

## Abstract

**Background:**

Hospital IT outages severely disrupt clinical workflows and use of electronic medical records, threatening patient safety and operational continuity. Traditional disaster response training faces limitations, including high resource requirements, restricted repeatability, and inability to be conducted without interrupting 24/7 hospital operations. Digital twin technology enables realistic, repeatable simulation training in virtual environments, avoiding operational disruption.

**Objective:**

This study developed and implemented a digital twin–based virtual hospital platform for level 1 IT outage disaster response training and evaluated its feasibility through quantitative performance metrics and participant survey feedback.

**Methods:**

A digital twin–based virtual hospital platform modeling 317 clinical spaces and 6 building entrances of Yongin Severance Hospital, South Korea, was developed to simulate a hospital information system failure (code white level 1 IT outage) with 7 patient cases of varying complexity levels, covering complete outpatient workflows from registration to payment. A total of 60 multidisciplinary participants (physicians, nurses, laboratory technicians, pharmacists, and administrative staff) were recruited through purposive sampling from clinical departments and support services directly involved in outpatient IT outage response. Emergency prescription and patient information lookup systems were integrated into the training. Performance evaluation included scenario completion rates, prescription accuracy, completion times, and operational readiness scores. Training outcomes were compared with 2023 conventional training records from the same institution using descriptive metrics. Open-ended survey responses were analyzed using structured content summarization with text mining and word cloud techniques. A 7-item operational readiness checklist was assessed by a panel of 5 training facilitators to evaluate system functional completeness.

**Results:**

In July 2024, a total of 60 multidisciplinary participants completed the training exercise. All 7 patient scenarios achieved 100% completion rates, with perfect accuracy in medical billing concordance and prescription entry timeliness. Scenario completion times ranged from 25 to 47 minutes, with variations reflecting testing wait times and workflow complexity. The overall operational readiness score was 80% (with 70% for digital twin platform operational readiness and 90% for emergency prescription program operational readiness). Training reduced resource consumption by 70 minutes compared to the 2023 conventional training approach, decreasing full-time equivalent requirements from 0.072 to 0.038. Open-ended survey feedback yielded 5 content categories (target extensions, mock training ideas, drug and prescription system improvement, operations and evaluation systems, and process improvement), with “realism,” “collaboration,” and “prescription” as the most frequent keywords.

**Conclusions:**

This proof-of-concept study demonstrates that a simulation-oriented digital twin platform can support multi-department IT disaster response training with complete workflow execution. Identified technical gaps in user permissions and prescription classification provide a concrete development road map for institutional deployment.

## Introduction

Modern health care delivery depends heavily on hospital information systems (HISs) for core processes, including appointment scheduling, prescription management, diagnostic testing, and billing [1]. This dependence makes IT system failures a critical threat to both patient safety and care continuity [2]. A 20-year analysis of acute care disruptions in Dutch hospitals documented 39 IT outage incidents that resulted in actual emergency care disruptions [3]. Of these, 85% originated from internal network and software errors, representing primary IT failures within hospital systems. While 92% (n=36) were resolved within hours, every incident required emergency department closure. Among these cases, 69% also suspended operating room operations, and 5% necessitated patient evacuation to external facilities. Most notably, 44% of incidents occurred simultaneously across multiple hospital sites, showing that IT failures can go beyond single institutions to affect entire regional health care systems. More recently, the 2024 Change Healthcare ransomware attack affected 190 million Americans, halting claims processing across thousands of organizations and forcing health care organizations to revert to paper-based workflows for weeks to months [4].

Traditional IT outage response training has relied predominantly on one-time field-based scenario exercises with limited participant numbers in a constrained environment [5]. This approach faces substantial practical limitations, lowering its potential to be developed into repeatable, multi-session training programs. Disaster response training inherently requires significant time and financial resources, and hospitals’ continuous operations hinder the execution of complete fully planned exercises [6]. For example, one study conducted a simulation training exercise at a German hospital facility involving 20 intensive care unit nurses responding to a hypothetical cyberattack on patient monitoring equipment [7]. The training used actor patients and monitor systems functioning identically to actual equipment in ward-like conditions. Nurses monitored vital signs and determined appropriate medication administration for 3 patients each while progressively exposed to cyberattack scenarios involving manipulated monitor data. While this approach demonstrated value in observing real-time behavioral responses, only approximately 60% of participants recognized equipment anomalies, indicating substantial room for improvement. A recent systematic review of full-scale simulation exercises for hospital disaster preparedness confirmed that, while such exercises improve staff response capabilities, their infrequent scheduling and high resource demands remain persistent barriers to widespread adoption [8].

Notably, digital twin technology offers a potential solution to the limitations of traditional approaches to disaster response training. Digital twin technology reproduces real-world environmental elements in a virtual space, allowing for simulation-based prediction and analysis of specific phenomena and their outcomes [9]. Originally developed for industrial and manufacturing applications, recent advances in high-performance computing, real-time data integration, and artificial intelligence have expanded the technology’s scope to complex socioenvironmental systems such as tsunami prediction and disaster response strategy development [10]. Digital twin technology has also gained recognition as a promising approach to securing and strengthening the resilience of health care systems, characterized by intricate patient flows and operational interdependencies [11]. For example, the HospiT’Win system offers a digital twin framework that replicates hospital operations in a virtual space, enabling real-time patient pathway tracking and prediction [12]. This system enables health care professionals to monitor patient pathway data and conduct scenario analyses for unexpected situations with a view to establishing appropriate response strategies. The framework also stresses the importance of integrating theoretical models with practical simulations and maintaining regular implementation schedules to build crisis response capabilities [13]. A recent meta-review of digital twin applications in health care reported growing implementation across operational and clinical domains but identified substantial challenges in data integration, validation, and scalability that remain unresolved [14].

Despite these developments and their potential to address the above-mentioned constraints of disaster response training, previous attempts at applying digital twin technology to health care crisis preparedness have revealed significant limitations. For example, one study constructed a virtual hospital network environment with a hybrid simulation model to analyze the quantitative impacts of ransomware attacks on medical imaging systems, including equipment operation suspension and security-related processing delays [15]. However, pilot implementation showed that the model failed to fully capture actual variations in clinical practice, process inconsistencies, and delayed postevent documentation, limiting both clinical applicability and generalizability.

This study addresses the repeatability limitations and resource requirements of conventional IT outage response training by implementing a digital twin–based virtual hospital platform. We designed and conducted code white (level 1 IT failure) simulation training that replicated complete outpatient workflows from registration to payment and evaluated both quantitative performance metrics and participant survey feedback to assess platform feasibility and future expansion potential.

## Methods

### Study Design

This study conducted disaster response simulation training at Yongin Severance Hospital, Yonsei University, an academic medical center designated as a digital health innovation hospital in South Korea [16], using a digital twin–based virtual hospital platform to simulate a code white (level 1 IT system failure) scenario. The study proceeded through four phases: (1) virtual hospital platform development, (2) IT outage response scenario design, (3) training operation with participation from multiple clinical departments and support staff, and (4) training outcome and participant feedback analysis. The implemented simulation used 7 patient scenarios reflecting outpatient care processes, enabling us to assess quantitative indicators, including completion rates and time requirements. Following training completion, we analyzed participant experiences (collected through surveys) using text mining and word cloud techniques for survey data assessment. Through this approach, we explored platform improvement directions and future expansion possibilities while validating the feasibility and effectiveness of the digital twin–based IT outage response training. The insights we gained were used to propose a new model for disaster response training.

### Digital Twin Platform Implementation

**Figure 1:**
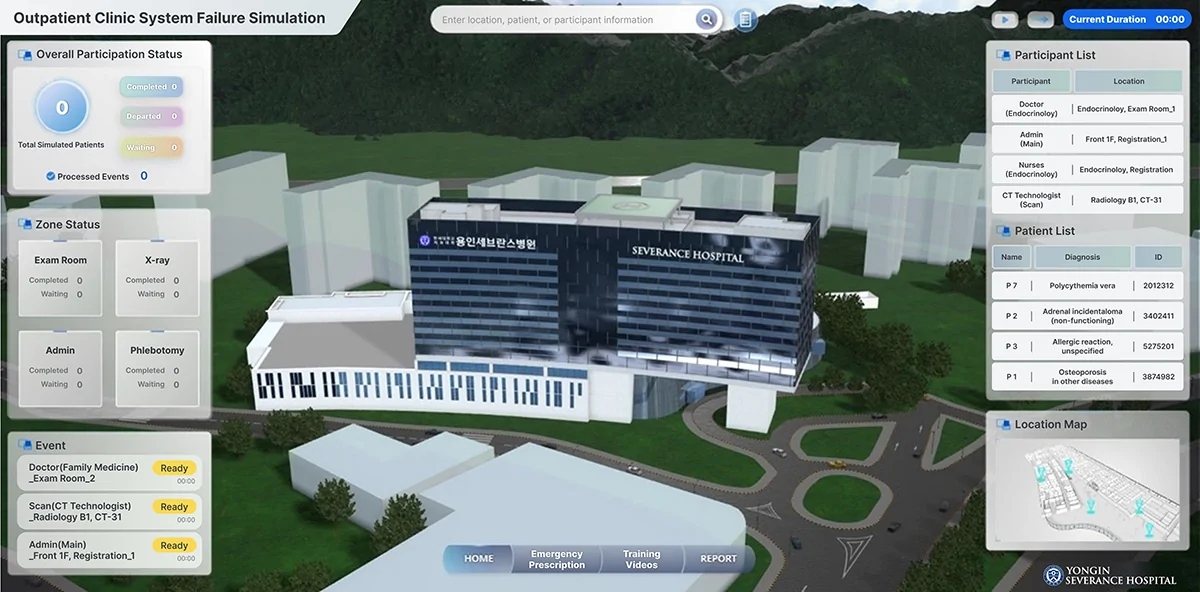
Real-time monitoring dashboard of the digital twin–based virtual hospital platform during IT outage simulation training. The dashboard displays real-time patient locations, departmental progress, and task completion rates across simulated outpatient workflows.

The digital twin–based virtual hospital platform was designed to replicate the actual outpatient environment, incorporating 317 clinical spaces and 6 building entrances (Figure 1). The platform was developed as a web-based application accessible through standard desktop computers, allowing for simultaneous multiuser access during training sessions. Detailed platform technical specifications, including the 9 development modules, system architecture, and 2024 updates, are provided in Multimedia Appendix 1.

For the training exercise, all participants gathered in the hospital’s computer room, where each was assigned a dedicated workstation. The digital twin application ran on the hospital’s local network infrastructure, with participants logging in through individual user credentials corresponding to their assigned roles (eg, physician, nurse, administrative staff, and pharmacist). This centralized physical arrangement facilitated real-time coordination and immediate troubleshooting during the simulation. The training participant organizational structure is shown in Figure S1 and Table S1 in Multimedia Appendix 1. The 9 development modules and their specifications are listed in Table S2 in Multimedia Appendix 1.

This simulation-oriented digital twin platform used preprogrammed patient avatars that entered through designated building entrances at preset times based on outpatient appointment schedules rather than incorporating real-time sensor data or live patient flow integration. The simulation engine adjusted patient movement speeds according to age and severity parameters. Escalator and elevator functions were incorporated to enable realistic interfloor movement patterns matching actual hospital workflows.

**Figure 2:**
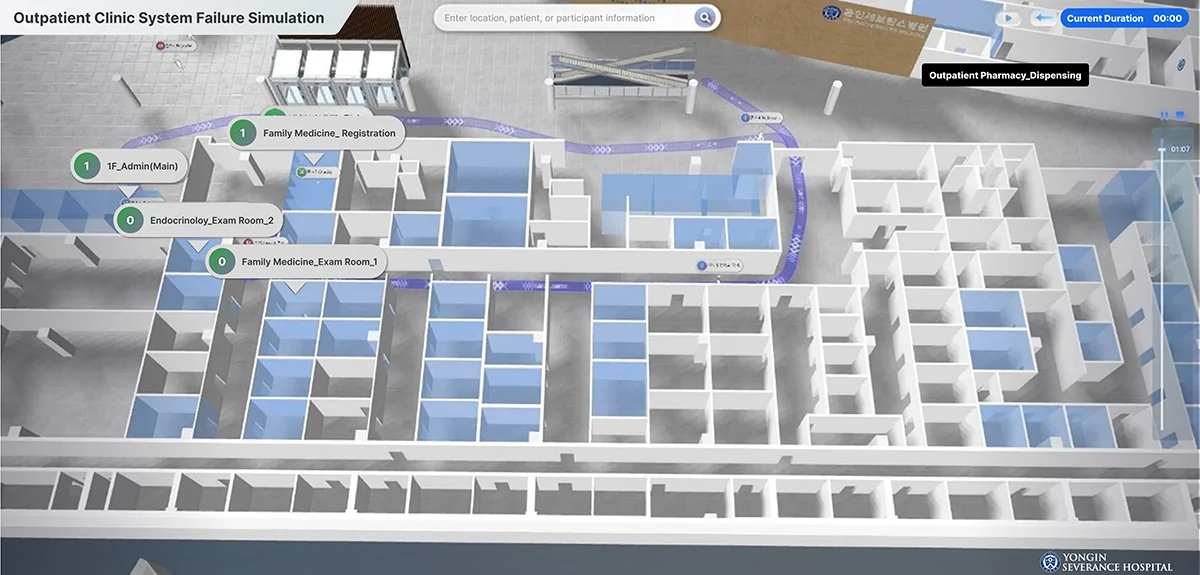
A 3D visualization of patient flow pathways within the digital twin–based virtual hospital platform replicating Yongin Severance Hospital, South Korea. The platform modeled 317 clinical spaces and 6 building entrances; participants navigated through the third-person view shown.

Each participant’s interface displayed a third-person view with an integrated dashboard showing real-time patient positions, departmental progress, and task completion rates (Figure 2). This allowed participants to track both individual patient scenario progression and overall training status simultaneously. The dashboard interface was role specific, displaying relevant information based on each user’s assigned department and responsibilities.

### Training Scenario Development and Operation

The training scenario was designed to simulate a hospital-wide level 1 IT outage (code white) with complete HIS paralysis due to potential hacking or program deployment errors. The scenario assumed that the order communication system and electronic medical records (EMRs) would be completely suspended, whereas network and internal communication infrastructure would remain operational. Under these conditions, emergency prescription programs based on internal messaging systems and rapid patient information lookup systems were designated as alternative means for clinical operations until system recovery.

Seven simulated patient scenarios were designed by complexity level (high, medium, and low) reflecting clinical department, visit type, and presenting symptoms. Scenario design was informed by the typical daily outpatient volume of 3500 visits to provide operational context. The scenario development process, including clinical validation procedures and complexity level criteria, is detailed in Table S3 in Multimedia Appendix 1). Each simulated patient completed the entire outpatient process of registration, consultation, testing, prescription, and payment on the virtual hospital platform, with detailed elements including patient movement paths and waiting times also reflected.

Participants were recruited through purposive sampling of staff directly involved in outpatient IT outage response at Yongin Severance Hospital. Department heads from clinical, nursing, laboratory, radiology, pharmacy, and administrative divisions were asked to identify personnel whose roles would be affected during a hospital-wide IT system failure. Multidisciplinary personnel were enrolled across 7 functional groups: administrative support, nursing support, clinical support, laboratory testing support, imaging support, pharmacy team, and patient services support. No formal sample size calculation was performed as the study aimed to include all relevant staff for a full-scale simulation exercise rather than test a specific hypothesis. All identified personnel agreed to participate; there were no refusals or nonresponses. The training was conducted as a single session on July 3, 2024. All participants received pretraining instruction on digital twin platform access and use of emergency prescription programs and rapid lookup systems based on internal messaging. Details of the 2 pretraining education sessions and the emergency prescription program development are provided in Multimedia Appendix 1. On training day, participants assembled at the training venue and completed real-time training on the platform according to assigned roles. During training, patient locations, departmental progress status, and task completion rates were monitored in real time through the dashboard.

### Data Collection and Analysis

Following training, quantitative data, including completion rates and time requirements for each patient scenario, were collected through the digital twin platform’s recorded logs. Official hospital documents, including IT outage response manuals and the previous year’s (2023) disaster response training records, were also collected. Operational readiness was assessed by a panel of 5 training facilitators (2 IT specialists, 1 nursing administrator, 1 clinical faculty member, and 1 emergency management coordinator) using a 7-item checklist covering system availability, user access functionality, prescription processing, billing accuracy, scenario continuity, emergency broadcast operations, and interface usability (Table S4 in Multimedia Appendix 1). Panel members independently scored each item as “pass” or “fail” based on direct observation during the training exercise, and the overall operational readiness score was calculated as the percentage of items rated as “pass.” Full-time equivalent (FTE) requirements were calculated as the number of participants multiplied by total training duration in hours divided by annual working hours per employee (2080 hours based on 8 hours per day, 5 days per week, 52 weeks per year). Detailed FTE calculation parameters and a comparison of the 2 training approaches are provided in Tables S5 and S6 in Multimedia Appendix 1. To compare resource efficiency between the 2024 digital twin–based training and the 2023 conventional training, we performed a descriptive comparison of aggregated training duration and FTE values as the 2023 records were available only as institutional summary documentation rather than individual-level data. The overall training execution workflow is illustrated in Figure S2 in Multimedia Appendix 1.

Open-ended survey data were collected immediately following the training session on July 3, 2024, using a structured paper-based feedback form distributed to all 60 participants who had provided prior consent. The survey consisted of a single open-ended item (Table S7 in Multimedia Appendix 1), and all 60 participants returned completed forms (100% response rate). Responses were classified into 7 groups (administrative support, nursing support, clinical support, laboratory testing support, imaging support, pharmacy team, and patient services support). Two researchers (SAB and YJK) independently read all responses and generated initial descriptive codes, which were organized in a spreadsheet and grouped into similar categories through discussion [17]. Text mining and word cloud techniques were applied in parallel focusing on core statements aligned with the research objective of “training advancement” to increase analysis validity and derive practical improvement measures.

### Ethical Considerations

This study received approval from the institutional review board of Yongin Severance Hospital (9-2026-0044). All participants provided written informed consent before training participation. No patient data were used in this study; all scenarios were based on simulated patients. This study adhered to the principles of the Declaration of Helsinki.

## Results

### Overview

The disaster response simulation training was conducted at Yongin Severance Hospital on July 3, 2024. This training, conducted without prior rehearsal, implemented simulation replacing core IT-based medical services, including consultation, prescription, testing, and payment during medical information system (order communication system and EMR) suspension. A total of 60 participants from multiple clinical departments and support services organized into 7 multidisciplinary groups (outpatient nursing teams, laboratory services, radiology, pharmacy, and administrative support) completed all 7 simulated patient scenarios (Table 1). The 7 simulated patient scenarios were structured according to difficulty level (high, medium, and low), with the complete process of registration, consultation, testing, payment, and pharmacy dispensing performed within the virtual environment, reflecting actual outpatient care flow.

**Table 1:** Characteristics of the 7 simulated outpatients used in the digital twin–based IT outage (code white level 1) disaster response simulation training^a^.

Patient ID	Difficulty level	Department	Hi-pass (yes or no)^b^	Visit type	Presenting symptoms
Patient 1	High	Endocrinology	No	First visit	Sudden increase in blood pressure, palpitations, and excessive sweating
Patient 2	High	Hematology and oncology	Yes	Follow-up	Persistent cough and dyspnea; referred from another hospital
Patient 3	Medium	Endocrinology	No	First visit	Recently feeling weak; concerned about possible osteoporosis due to aging
Patient 4	Medium	Family medicine	No	First visit	Sneezing, itchy eyes, and nasal congestion
Patient 5	Medium	Family medicine	Yes	Follow-up	Obesity and gradual weight gain
Patient 6	Low	Family medicine	No	Follow-up	Lower back pain and discomfort in right upper abdomen with tenderness
Patient 7	Low	Hematology and oncology	Yes	First visit	Anemia, dizziness, fatigue, and lightheadedness

^a^Patient scenarios were stratified by complexity level (high, medium, and low) reflecting clinical department, visit type, and presenting symptoms.

^b^Hi-pass: the hospital’s automated billing system for one-stop outpatient payment.

**Figure 3:**
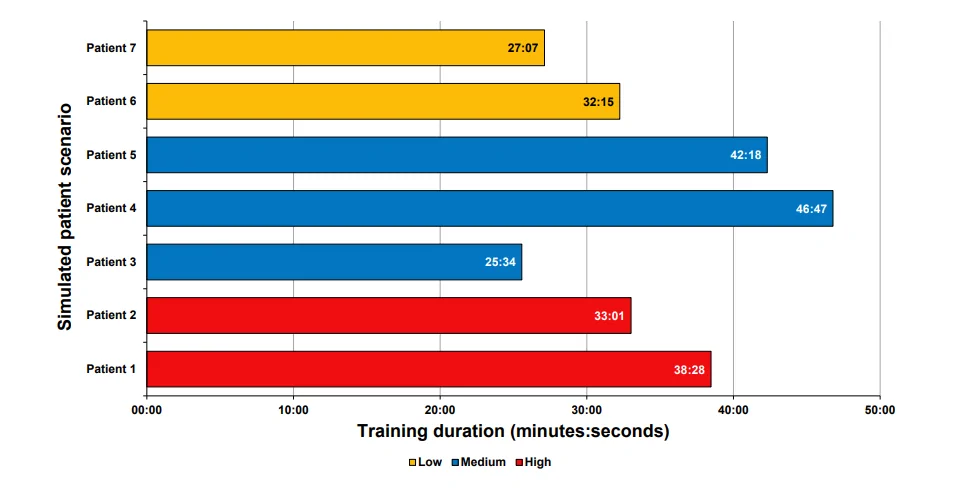
Training time required to complete each of the 7 simulated outpatient scenarios. Patients were stratified by complexity level (high, medium, and low).

Training time varied according to patient scenario contents, interdepartmental movement routes, and prescription entry procedures, with the medium-difficulty patient group having the longest execution time (patient 4: 46 minutes 47 seconds; patient 5: 42 minutes 18 seconds; Table 2). Meanwhile, the high-difficulty scenarios averaged approximately 35.7 (SD 3.9) minutes, and low-difficulty scenarios were completed within 29.7 (SD 3.6) minutes. In particular, one of the medium-difficulty simulated patients (patient 4) showed extended execution time due to testing wait times and repeated prescription entry (Figure 3). These results suggest that differences in clinical flow and task complexity by patient type can directly affect training time requirements.

**Table 2:** Training performance rates and time required to complete each of the 7 simulated outpatient scenarios^a^.

Patient ID	Difficulty level	Training duration	Scenario^b^
Patient 1	High	38 min 28 s	B1_Entrance 2 (0 min)→endocrinology reception (27 s)→administrative office main counter 1 (1 min 49 s)→endocrinology reception (50 s)→consultation room 1 (9 min 55 s)→endocrinology reception (4 min 23 s)→department of laboratory medicine—phlebotomy (5 min 15 s)→reception (29 s)→department of radiology—CT^c^ room 31 (6 min 15 s)→F2_Entrance 1
Patient 2	High	33 min 1 s	F1_Entrance 1 (0 min)→administrative office main counter 1 (2 min 3 s)→hematology and oncology reception (1 min 30 s)→consultation room 1 (7 min 32 s)→outpatient pharmacy reception (3 min 24 s)→administrative office main counter 1 (1 min 42 s)→F1_Entrance 3
Patient 3	Medium	25 min 34 s	B1_Entrance 1 (0 min)→administrative office main counter 1 (1 min 55 s)→endocrinology reception (2 min 30 s)→consultation room 1 (6 min 20 s)→endocrinology reception (2 min 52 s)→department of radiology—CT room 31 (6 min 43 s)→B1_Entrance 1
Patient 4	Medium	46 min 47 s	B1_Entrance 1 (0 min)→family medicine reception (1 min 36 s)→administrative office main counter 1 (1 min 59 s)→family medicine reception (1 min 42 s)→consultation room 1 (9 min 52 s)→family medicine reception (7 min 17 s)→consultation room 1 (1 min 14 s)→reception (1 min 32 s)→department of laboratory medicine—phlebotomy (1 min 31 s)→reception (1 min 23 s)→outpatient pharmacy reception (36 s)→F1_Entrance 2
Patient 5	Medium	42 min 18 s	B1_Entrance 1 (0 min)→family medicine reception (26 s)→administrative office main counter 1 (3 min 28 s)→family medicine reception (30 s)→consultation room 1 (11 min 21 s)→family medicine reception (9 min)→outpatient pharmacy reception (1 min 2 s)→administrative office main counter 1 (1 min 38 s)→F2_Entrance 1
Patient 6	Low	32 min 15 s	F1_Entrance 1 (0 min)→family medicine reception (1 min 38 s)→administrative office main counter 1 (2 min 27 s)→family medicine reception (1 min 32 s)→consultation room 1 (11 min 45 s)→family medicine reception (6 min 57 s)→outpatient pharmacy reception (31 s)→F1_Entrance 3
Patient 7	Low	27 min 7 s	F1_Entrance 1 (0 min)→hematology and oncology reception (3 s)→administrative office main counter 1 (4 min 16 s)→hematology and oncology reception (1 min 3 s)→consultation room (3 min 32 s)→outpatient pharmacy reception (4 min 46 s)→department of laboratory medicine—phlebotomy (3 min 59 s)→reception (53 s)→outpatient pharmacy reception (3 min 40 s)→F1_Entrance 1

^a^Completion rates and times were derived from the platform’s automatically recorded logs.

^b^B1_Entrance: entrance located on Basement Floor 1; F1_Entrance and F2_Entrance: entrances on Floor 1 and Floor 2, respectively.

^c^CT: computed tomography.

The comparison of medical billing between emergency prescription programs and HIS programs showed full concordance (100%); prescription and test result entry timeliness (100%) and scenario consecutive execution rate (100%) also demonstrated full completion quality. The emergency response broadcast system (code white) operation status was also confirmed at 100%. In contrast, digital twin platform operational readiness was rated at 70%, reflecting some technical limitations, including insufficient user permission–based menu structure and inadequate interface intuitiveness. While the emergency prescription program was rated at 90%, needs for improvement in prescription classification item display and target patient designation functions were mentioned (Table 3).

**Figure 4:**
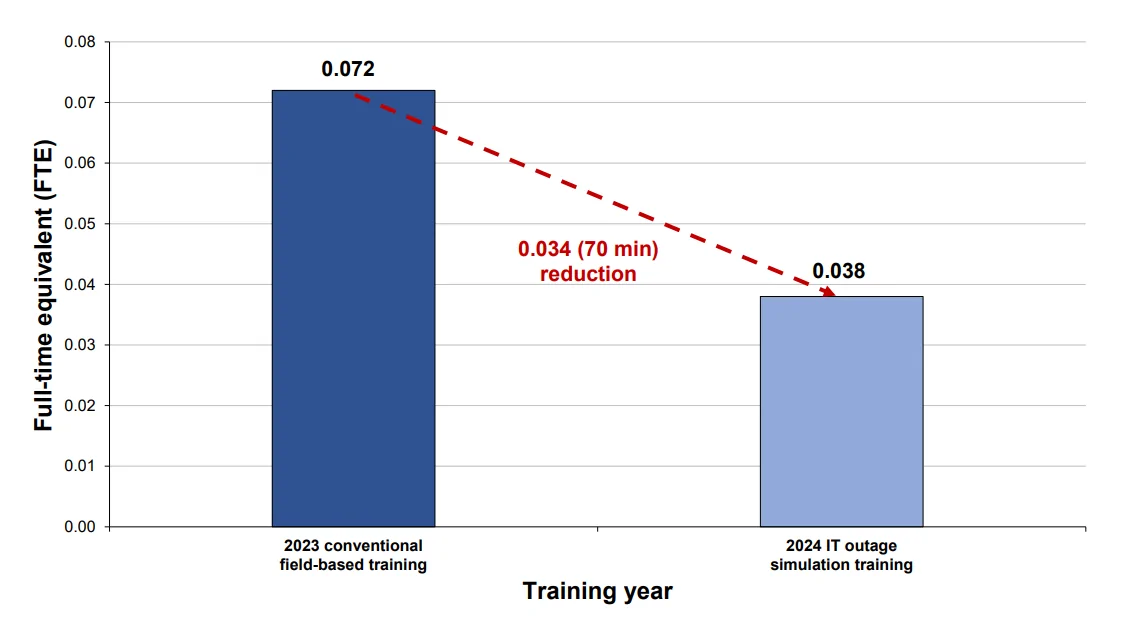
Training resource savings comparing the 2024 digital twin–based IT outage disaster response simulation training with the 2023 conventional field-based training. Values are expressed as full-time equivalent (FTE). FTE was calculated as the number of participants multiplied by training duration in hours divided by annual working hours per employee (2080 hours based on 8 hours per day, 5 days per week, 52 weeks per year).

Resource efficiency analysis showed that the digital twin–based training reduced training time requirements by approximately 70 minutes compared to the previous year’s conventional training approach. This translated to a 0.034 reduction in FTE staffing from 0.072 in 2023 to 0.038 in 2024 (Figure 4).

**Table 3:** Quantitative evaluation results for the 6 simulation performance indicators^a^.

Evaluation item	Completion rate (%)	Summary of findings
Accuracy of medical billing	100	Emergency prescription records matched HIS^b^ data.
Timeliness of order entry and results	100	All simulated patient prescriptions and test results were entered as planned.
Scenario execution continuity	100	All 7 patient scenarios were completed without interruption.
Digital twin platform operational readiness	70	Some interface elements required role-based access control and UI^c^ optimization.
Emergency prescription program operational readiness	90	Minor improvements needed for order classification and assigned patient search functions.
Code broadcast and control center operations	100	Code white announcement and emergency messaging were delivered on time.

^a^Indicators were rated by a panel of 5 training facilitators using a “pass” or “fail” criterion based on direct observation of the training exercise.

^b^HIS: hospital information system.

^c^UI: user interface.

### Open-Ended Survey Feedback

Structured content summarization of participant responses to a single open-ended question (administered immediately after training) identified 5 content categories. Each category is described below; the full set of categorized responses is summarized in Table 4.

**Table 4:** Content categorization of simulation enhancement ideas derived from open-ended survey responses collected from 60 multidisciplinary hospital staff members immediately following the digital twin–based IT outage disaster response simulation training^a^.

Category	Advanced topic keywords
Target extensions	Inpatient simulation Emergency department simulation
Mock training ideas	Code red preparedness Evacuation of ventilated patients Fire evacuation drill Radiation exposure training
Drug and prescription system improvement	Order classification refinement Anticancer medication simulation Verification of external prescriptions for outpatients
Operations and evaluation systems	Development of evaluation metrics
Process improvement	Outpatient workflow optimization Inpatient process improvement

^a^Responses were classified into 5 content categories through structured content summarization.

Participants expressed positive perceptions regarding training realism and platform effectiveness. Text mining and word cloud analysis of open-ended responses revealed that the most frequently mentioned keywords were “realism,” “collaboration,” and “prescription,” indicating high engagement with the simulation environment and interdepartmental coordination processes.

Regarding future training directions, participants proposed expansion to high-risk scenarios, including inpatient simulation, emergency department simulation, evacuation of ventilated patients, fire evacuation drills, and radiation exposure training. Additional suggestions included code red preparedness training and anticancer medication simulation. From an operational perspective, participants recommended outpatient workflow optimization, development of standardized evaluation metrics, and refinement of order classification functions to better mirror actual EMR systems. Specific technical improvements included verification mechanisms for external prescriptions and enhanced patient search functions within the emergency prescription program.

## Discussion

### Principal Findings

This paper reports the implementation of a simulation-oriented digital twin platform for IT outage disaster response training at a single academic medical center, engaging 60 participants across multiple clinical departments to simulate complete outpatient workflows during a level 1 IT system failure. The achievement of 100% completion rates across all 7 patient scenarios, combined with perfect accuracy in medical billing (100%) and prescription entry timeliness (100%), demonstrates that digital twin technology can reliably reproduce complex clinical processes in a virtual environment. These quantitative results support the feasibility of simulation-oriented digital twin platforms for disaster response training as a proof of concept, with specific technical refinements identified for future development.

The clinical significance of these findings extends beyond simple task completion metrics. Electronic health record downtime creates substantial patient safety risks, with documented cases of medication errors, delayed treatments, and compromised clinical decision-making during system unavailability [5]. Recent systematic reviews emphasize that health care organizations must develop robust business continuity protocols specifically for prolonged IT outages as recovery times have increased substantially in recent years [18]. Our training platform’s capacity to simulate interconnected workflows across departments prepares staff not just for isolated system failures but for the system-wide disruptions that characterize modern IT disasters, where cascading effects can overwhelm entire regional health care systems.

The 70-minute reduction in training duration (compared to traditional training approaches), translating to a 0.034 FTE decrease (from 0.072 to 0.038), should be interpreted as a descriptive operational comparison rather than a causally attributable efficiency gain given that all 60 participants in 2024 were identical to the 2023 cohort (100% overlap; potential learning effect) and the virtual format inherently eliminated physical transit time. Nevertheless, the elimination of physical redeployment represents an inherent advantage of digital twin–based training as it preserves clinical staff availability during training. In health care environments, where every hour of clinical staff time directly impacts patient care capacity, this efficiency gain enables institutions to conduct more frequent training iterations without compromising operational capacity. Recent digital twin applications in health care have demonstrated similar operational improvements: Siemens Healthineers reported 19-minute reductions in computed tomography and magnetic resonance imaging wait times with 32% and 26% capacity increases, respectively, alongside significant overtime reductions [19]. Studies of hospital digital twin implementations show that workflow optimization through virtual modeling can reduce bottlenecks while simultaneously improving staff satisfaction and reducing burnout [20]. Our resource efficiency data suggest that digital twin training platforms can achieve comparable operational optimization while simultaneously strengthening disaster preparedness—a dual benefit that traditional training approaches cannot match.

The operational readiness scores of 70% for the digital twin platform and 90% for the emergency prescription program, assessed by a 5-member panel using a 7-item checklist (Table S4 in Multimedia Appendix 1), provide concrete targets for system refinement. Participant feedback specifically identified the absence of EMR prescription classification features (“L,” “P,” and “R” coding for laboratory, pharmacy, and radiology orders) as the primary operational constraint. This finding aligns with broader implementation science literature demonstrating that successful health IT adoption requires precise replication of existing clinical workflows and careful attention to user experience design [21]. The identified need for role-based access control and department-specific interfaces reflects fundamental principles in digital twin design: virtual replicas must mirror not just physical spaces and processes but also the cognitive and operational patterns of actual clinical environments [22]. These technical limitations, rather than representing failures, highlight the specific development pathways toward production deployment.

The scale of participation (60 staff members across 7 functional groups) distinguishes this implementation from prior digital twin applications in health care [15], which have predominantly focused on operational optimization rather than disaster response training. Digital twin technology has proven effective in manufacturing and aerospace engineering for testing scenarios too dangerous or expensive to implement in reality, and health care applications are increasingly adopting similar approaches [23]. By extending this capability from workflow optimization to disaster preparedness, our study demonstrates that digital twins can serve dual functions: improving daily operations while simultaneously building organizational resilience against catastrophic failures. This convergence of operational efficiency and crisis preparedness suggests a potential shift in how health care organizations approach both quality improvement and disaster readiness.

Health care IT disasters demonstrate why crisis-specific training is essential. The 2024 Change Healthcare ransomware attack affected 190 million Americans, halted claims processing across thousands of organizations, and forced health care organizations to exhaust personal funds to maintain operations [4]. Health care facilities experienced significant operational disruptions lasting weeks to months, forcing health care organizations to revert to paper-based workflows and, in severe cases, requiring complete system restoration. These events affect entire integrated systems simultaneously (not isolated departments), requiring coordinated multi-department response capabilities that existing training approaches fail to develop.

Notably, existing digital twin implementations focus on operational optimization rather than crisis response. Digital twins can optimize clinical operations by analyzing workflows and resource allocation under normal conditions, supporting real-time monitoring and predictive analytics [8,14]. Conventional disaster training faces different constraints: full-scale exercises require 2 to 8 hours and extensive physical setup, limiting training frequency [24]. Although a Swiss pharmacy study showed task completion improving from 69% to 84% following conventional disaster training, 4-month intervals were required between training sessions [25].

Our platform addresses these limitations through 3 design features. The first is crisis-oriented simulation. Unlike operational optimization tools, our system trains staff for complete system absence rather than process improvement. The second is multi-department integration. Our platform simulated coordinated workflows across administrative, clinical, laboratory, pharmacy, and imaging departments, reflecting the cascading interdependencies of real IT disasters. The third is scalability. Accommodating 60 participants simultaneously without physical setup enables weekly rather than quarterly training sessions, supporting the frequent practice necessary for crisis skill retention.

Finally, our implementation demonstrates rapid deployment potential that previous approaches have not achieved. The Saskatchewan Hospital digital twin integration in Canada focused on emergency department optimization with continuous stakeholder engagement over extended time frames [26]. Our approach of deploying a functional training platform within months and conducting full-scale exercises with 60 participants demonstrates that digital twin technology has matured sufficiently for rapid deployment in urgent crisis preparation contexts, suggesting that disaster preparedness platforms can scale across health care systems more readily than gradual implementation approaches [27].

This study demonstrates that digital twin platforms can transform health care disaster preparedness from infrequent, resource-intensive exercises to routine, scalable training. By safely simulating complete system failures with 60 participants across multiple departments, we show that crisis-specific training can overcome historical limitations preventing frequent, realistic preparation. Our findings suggest that digital twin technology offers a practical pathway for coordinated crisis response training, with potential extending beyond IT disasters to other high-stakes scenarios requiring multi-departmental coordination.

### Limitations

First, this study’s single-institution implementation limits the generalizability of the findings to different health care settings. However, major digital twin initiatives similarly began with single-site pilots before widespread adoption [28]. Additionally, the 100% participant overlap between the 2023 and 2024 cohorts introduces a learning effect that confounds the FTE comparison; future multisite studies with independent participant groups are needed. Implementation science frameworks emphasize phased evaluation, with proof-of-concept studies identifying barriers before scaling. Our study establishes feasibility for disaster-specific digital twins, offering a foundation for multi-institutional validation.

Second, the results showing 70% digital twin platform operational readiness and 90% emergency prescription program operational readiness demonstrate that the platform requires further refinement. However, the absence of EMR prescription classification and role-based access control represents an implementation gap rather than a design flaw. Early electronic health record implementations faced similar challenges requiring iterative development [29]. These are solved problems in commercial systems: our identification of specific requirements provides a concrete development road map. We also acknowledge that the operational readiness assessment was not based on a validated usability instrument (eg, System Usability Scale or Post-Study System Usability Questionnaire); future studies should incorporate such instruments to capture individual-level usability perceptions.

Third, only 7 patient scenarios were implemented; thus, the simulation had limited variation in complexity. Training literature demonstrates that focused scenarios enable foundational skill development and recommends iterative introduction of complexity. Our scenarios established pragmatic range boundaries. Future iterations should incorporate scenarios common in actual cyberattacks: multiple simultaneous high-acuity patients, equipment failures, communication degradation, and incomplete information decision-making. The absence of these variables constrains the generalizability of our findings to real crisis conditions, where cognitive overload and resource exhaustion are defining challenges.

### Conclusions

This study demonstrates that digital twin–based virtual hospital platforms can effectively train staff for IT outage disaster response. Achieving 100% scenario completion validates functional feasibility as a proof of concept for crisis preparation training. Our findings suggest that simulation-oriented digital twin platforms may help address limitations in disaster preparedness training by enabling repeatable exercises without disrupting operations, although further validation using standardized usability instruments and higher-fidelity scenarios is needed. This approach has potential applications extending beyond IT outages to other disaster scenarios.

## Data Availability

The datasets generated or analyzed during this study are not publicly available due to institutional privacy policies but are available from the corresponding author on reasonable request.

## Appendix

Multimedia Appendix 1 Detailed platform technical specifications, training scenario development and clinical validation, operational readiness assessment methodology, full-time equivalent calculation, qualitative data collection and analysis, pretraining education program, and emergency prescription program.

## Abbreviations

EMR: electronic medical record
FTE: full-time equivalent
HIS: hospital information system
