# Supplementary material for: Digital Twin–Based Virtual Hospital Platform for IT Outage Disaster Response Training: Implementation and Evaluation Study
Source: JMIR Form Res. 2026 Aug 21;10:e93135. doi: 10.2196/93135 (PMC13545525; doi:10.2196/93135)
Supplement: Supplementary file 1 [file formative_v10i1e93135_app1.pdf]

SUPPLEMENTARY MATERIALS

Contents

Supplementary Methods

Platform Technical Specifications
Training Scenario Development and Clinical Validation
Operational Readiness Assessment Methodology
Full-Time Equivalent Calculation
Qualitative Data Collection and Analysis
Pre-Training Education Program
Emergency Prescription Program

Supplementary Tables

Table S1. Participant group composition and assigned training roles
Table S2. Platform development components and specifications
Table S3. Scenario complexity level criteria and characteristics
Table S4. The 7-item operational readiness assessment checklist
Table S5. FTE calculation parameters comparing the 2023 conventional field-based training and the 2024 digital twin-based training for IT outage disaster response
Table S6. Side-by-side comparison of the 2023 conventional field-based training and the 2024 digital twin-based training for IT outage disaster response
Table S7. Open-ended survey instrument administered to participants

Supplementary Figures

Figure S1. Training participant organizational structure
Figure S2. Training execution workflow

Supplementary Methods

Platform Technical Specifications

The digital twin-based virtual hospital platform was developed by IOI Soft (Seoul, Republic of Korea) over approximately four months (August–December 2023), with additional updates applied on June 19, 2024. The platform was built as a web-based application using a 3D modeling engine that rendered the physical environment of Yongin Severance Hospital, including 317 clinical spaces across multiple floors and 6 building entrances.

The system consisted of three primary components. The first was a 3D visualization engine responsible for rendering the hospital environment with real-time patient avatar movement, incorporating patient-specific movement speeds adjusted for age and clinical severity, as well as inter-floor transit via escalator and elevator functions. The second was a simulation control engine managing patient scenario progression, event triggers, and workflow sequencing through REST API interfaces. The third was a dashboard and reporting module that provided real-time monitoring of training status, patient positions, departmental progress, and automated post-training performance reports including per-patient completion times.

The platform operated on the hospital's local network infrastructure. During training, all 60 participants were physically assembled in the hospital's computer training room, each assigned a dedicated workstation. Participants logged in through individual credentials corresponding to their assigned professional roles (physician, nurse, administrative staff, pharmacist, laboratory technician, radiologic technologist, etc.). The training participant organizational structure is shown in **Supplementary Figure S1** and **Table S1**. This centralized arrangement allowed real-time coordination among training facilitators and immediate troubleshooting. The 9 development modules and their specifications are listed in **Supplementary Table S2**.

Supplementary Table S1. Composition of the 60 multidisciplinary participants and their assigned training roles

No.	Functional Group	Departments	Roles During Training
1	Administrative support	General Affairs Team, Administrative Office	Patient registration, billing verification, post-payment confirmation, external prescription printing
2	Nursing support	Outpatient Emergency Nursing Team (Parts 1 and 2)	Patient identification, order confirmation, inter-departmental referral coordination, next-visit scheduling

3	Clinical support	Endocrinology, Family Medicine, Hematology-Oncology	Diagnosis entry, order input (laboratory, imaging, pharmacy), clinical documentation on emergency record forms
4	Laboratory support	Dept. of Laboratory Medicine	Specimen receipt, order verification, test result upload to emergency prescription program
5	Imaging support	Dept. of Radiology, Nuclear Medicine Team	Imaging order confirmation, examination execution, consent form documentation, result upload
6	Pharmacy	Pharmacy Team (Outpatient Pharmacy)	Prescription verification, in-house medication dispensing, patient medication counseling
7	IT and platform support	Medical Information Technology Team	Platform operation, emergency prescription program management, troubleshooting, dashboard monitoring

A total of 60 participants across all groups completed the training exercise on July 3, 2024.

Supplementary Table S2. Platform development components and specifications

No.	Development Module	Specifications
1	UI design and planning	Interface layout design and user experience planning
2	3D building modeling	Hospital building reconstruction with multi-floor 3D modeling (317 clinical spaces, 6 entrances)
3	Simulation schema definition	Training and dashboard database schema design
4	REST API definition	Training and dashboard REST API specification
5	Participant registration	Role assignment (job title), name, training location (department), floor
6	Patient master registration	Patient name, registration number, departure time, diagnosis, age, clinical events
7	Participant interaction screens	Task input, query, scenario completion, and memo screens
8	Main display and dashboard	Training timer, patient movement visualization, wait status, event display, completion/waiting indicators
9	Training results report	Per-patient time breakdown, cumulative time, memos, training results, overall summary

Platform developed by IOI Soft (Seoul, Republic of Korea); initial development August–December 2023, with updates applied June 19, 2024.

Supplementary Figure S1. Training participant organizational structure

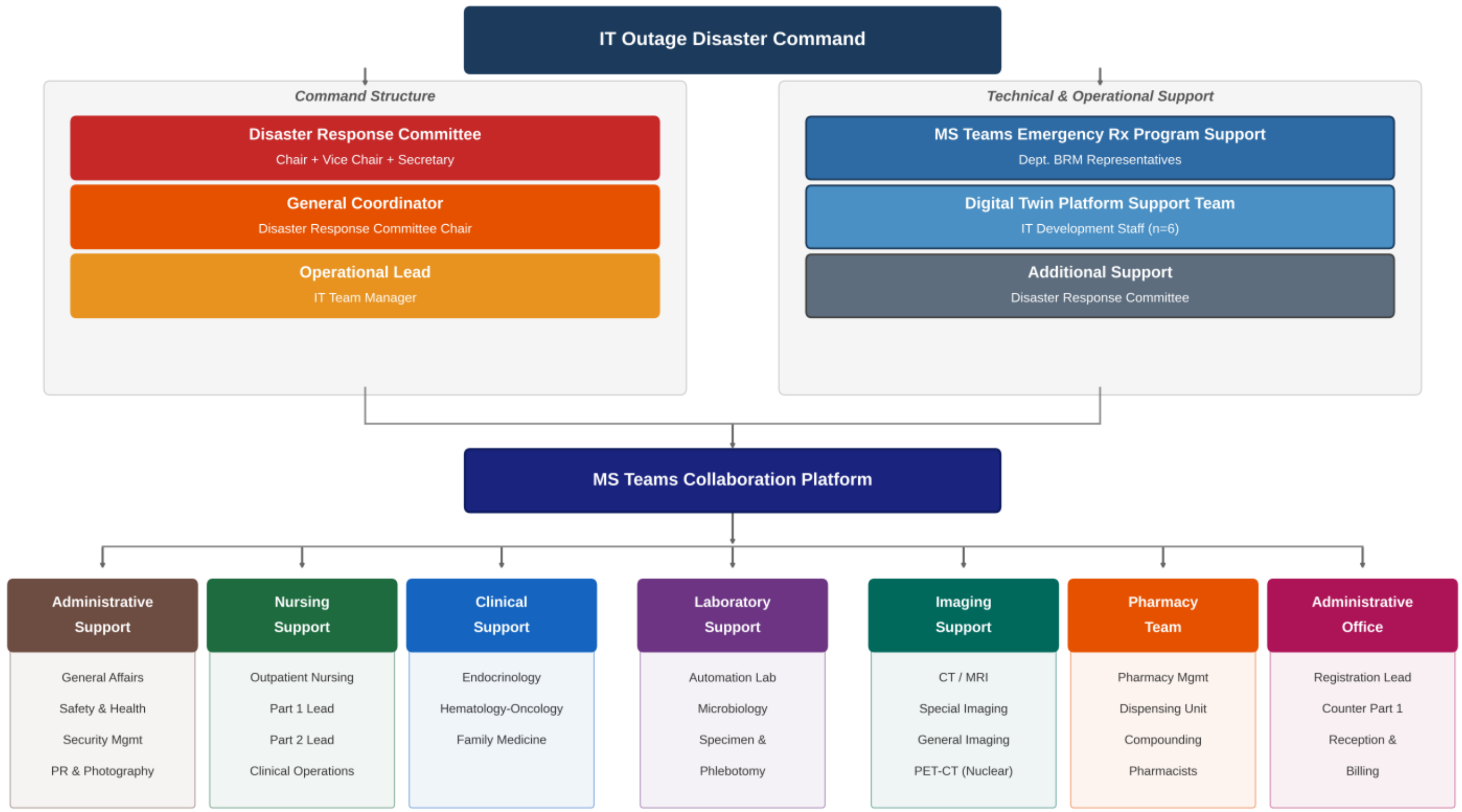

The organizational structure consisted of two parallel arms under the IT Outage Disaster Command. The Command Structure (left) included the Disaster Response Committee, General Coordinator, and Operational Lead. Technical and Operational Support (right) included the MS Teams Emergency Prescription Program Support, Digital Twin Platform Support Team, and Additional Support. All groups communicated via the MS Teams Collaboration Platform. Seven functional groups encompassing 60 participants carried out the simulation. Individual names have been removed for anonymization.

Prior to the July 2024 training, 15 platform updates were applied based on internal testing: scenario display error correction, training location pop-up verification, room list visualization improvement, selection-based room entry (replacing manual typing), event confirmation window redesign, progress status terminology updates, patient location arrival alerts, quiz response processing fixes, action checklist logging improvements, memo input screen adjustments, patient recognition enhancements, training completion notification pop-ups, age- and disease-specific walking speed calibration, training room display resolution optimization with GPU upgrades, and final report error verification.

Training Scenario Development and Clinical Validation

The training scenario was developed through a structured institutional process. The Disaster Response Subcommittee convened on June 25, 2024, with 16 members present, including the Disaster Response Committee Chair and the Administrative Director. Based on the 2024 institutional risk analysis—which identified communication and information system failures as the highest-risk categories—the committee resolved to conduct digital twin-based IT outage simulation training.

Departmental IT outage response manuals, which had been revised over three review cycles in 2023, formed the procedural basis for the scenarios. Clinical content was designed by the Medical Information Technology team in consultation with the three participating clinical departments (Endocrinology, Family Medicine, and Hematology-Oncology). Each simulated patient scenario was assigned a complexity level (High, Medium, or Low) based on three factors: (1) the number and type of orders required (laboratory, imaging, pharmacy), (2) the number of inter-departmental handoffs, and (3) whether the patient required new registration or was already in the system. The complexity criteria are summarized in **Supplementary Table S3**.

Supplementary Table S3. Scenario complexity level criteria and characteristics

Complexity	n	Departments	Order Types	Distinguishing Features
High	2	Endocrinology (1), Hematology-Oncology (1)	Laboratory (up to 16 items), imaging (CT, PET-CT), pharmacy	New patient registration required; multiple specimen types (serum, urine, plasma); external hospital referral
Medium	3	Endocrinology (1), Family Medicine (2)	Laboratory, imaging (DEXA, X-ray), pharmacy (in-house and external)	Mixed order types; in-house medication dispensing with reason codes; nuclear medicine referral
Low	2	Family Medicine (1), Hematology-Oncology (1)	Laboratory, pharmacy only	Fewer inter-departmental transfers; simpler order sets

Complexity was assigned based on: (1) number and type of orders, (2) number of inter-departmental handoffs, and (3) new versus existing patient registration status.

Clinical validity was established as follows. All patient presentations, diagnoses, and corresponding orders were based on common outpatient clinical presentations at the participating departments. Order sets—including specific laboratory test codes, imaging protocols, and medication names with dosages—were drawn directly from the hospital's actual order

communication system (OCS) master data. A pre-training explanatory session was held on June 27, 2024, where the scenarios were reviewed by 30 participants from all departments, and feedback was incorporated before finalization. No rehearsal of the actual training scenarios was conducted prior to the July 3 exercise.

Operational Readiness Assessment Methodology

Post-training performance was evaluated using a 7-item operational readiness checklist developed by the training operation team (Medical Information Technology team and Disaster Response Committee). This checklist was designed to assess whether each critical system component and workflow functioned as intended during the simulation, rather than to measure subjective user satisfaction.

Each item was assessed by a panel of 5 Medical Information Technology team members who served as training facilitators. Assessment was based on direct observation of the training process, review of platform-recorded logs, and cross-verification with emergency prescription program records. Items involving binary outcomes (e.g., billing concordance, scenario completion) were scored as 100% upon full concordance. For system functionality items (items 4 and 5), scores reflected the proportion of intended features that operated without issues. Specific assessment methods for each checklist item are described in **Supplementary Table S4**.

Supplementary Table S4. The 7-item operational readiness assessment checklist

No.	Checklist Item	Assessment Method
1	Medical billing concordance	Emergency prescription billing records compared against HIS data after system recovery.
2	Timeliness of order entry and result input	Verification of whether all prescriptions and test results were entered within designated timeframes per scenario protocol.
3	Simulated patient scenario continuity	Assessment of whether all patient scenarios completed the full registration-to-payment workflow without interruption.
4	Digital twin platform operational readiness	Evaluation of platform functionality based on the proportion of intended features that operated without issues, including user role-based menu access control and interface intuitiveness.
5	Emergency prescription program operational readiness	Evaluation of prescription program functionality, including L/P/R (laboratory/pharmacy/radiology) order classification display and designated patient search functions.
6	Emergency patient lookup program	Verification of patient information lookup via u-Severance emergency query system across all scenarios.

7	Code White broadcast and control center operations	Verification of hospital-wide Code White announcement broadcast timing and SMS emergency notification delivery to all designated personnel.

Each item was scored as pass or fail based on direct observation. Assessment conducted by a panel of 5 Medical Information Technology team members serving as training facilitators, based on direct observation, platform-recorded logs, and emergency prescription program records. HIS = hospital information system; L/P/R = laboratory/pharmacy/radiology. This assessment was not based on a validated usability instrument (e.g., SUS, PSSUQ).

This assessment was not based on a validated usability instrument such as the System Usability Scale (SUS) or the Post-Study System Usability Questionnaire (PSSUQ). The checklist was designed for internal institutional evaluation of system functional completeness during a proof-of-concept implementation. We acknowledge this as a limitation and recommend that future studies incorporate validated usability instruments administered to individual participants.

Full-Time Equivalent Calculation

Full-time equivalent (FTE) values were calculated to compare resource consumption between training years using the formula: FTE = (number of participants × training duration in hours) / annual working hours per employee. Annual working hours were based on the standard Korean labor framework (8 h/day × 5 days/week × 52 weeks/year = 2,080 h/year). The detailed parameters are provided in **Supplementary Table S5**.

Supplementary Table S5. FTE calculation parameters comparing the 2023 conventional field-based training and the 2024 digital twin-based training for IT outage disaster response

Parameter	2023 Conventional Training	2024 Digital Twin Training
Training format	Field-based exercise with physical deployment across actual hospital departments	Virtual simulation; all participants in computer training room
Participants	60	60
Duration breakdown	Briefing + physical deployment across departments + scenario execution + debriefing	Emergency prescription instruction + virtual simulation + u-Severance data entry + debriefing
FTE formula	(Participants × duration in hours) / annual working hours	(Participants × duration in hours) / annual working hours

Annual working hours	2,080 h (8 h/day × 5 days/week × 52 weeks/year)	2,080 h (8 h/day × 5 days/week × 52 weeks/year)

FTE = full-time equivalent. Actual FTE values and time savings are reported in the Results section of the main manuscript.

Three confounding variables should be considered when interpreting this comparison. First, the 2024 participant pool partially overlapped with the 2023 cohort; prior experience with the disaster protocol may have contributed to faster completion independent of the platform (learning effect). Second, the 2023 training involved physical movement across actual hospital departments, whereas the 2024 training was conducted entirely via virtual simulation, meaning that the time reduction partially reflects inherent elimination of physical transit and setup time. Third, while both years addressed the same disaster type with similar clinical workflows, exact task composition was not strictly controlled across years. The FTE comparison should therefore be interpreted as a descriptive operational comparison rather than a controlled measure of platform-attributable efficiency. A side-by-side comparison of the two training approaches is provided in **Supplementary Table S6**.

Supplementary Table S6. Side-by-side comparison of the 2023 conventional field-based training and the 2024 digital twin-based training for IT outage disaster response

Feature	Conventional Training (2023)	Digital Twin Training (2024)
Environment	Actual hospital departments with physical staff deployment across floors	Virtual hospital platform; all participants in a single computer room
Operational impact	Requires partial disruption; cannot be conducted during peak hours without affecting patient care	No disruption to clinical operations
Spatial constraints	Limited by physical space; coordination across departments required	No spatial constraints; full hospital environment replicated virtually
Scenario design	Limited by physical logistics and available personnel	Multiple patient scenarios with tiered complexity levels; expandable
Patient flow monitoring	Manual tracking only	Real-time 3D visualization with integrated dashboard
Prescription method	Paper-based manual prescription forms	MS Teams-based emergency prescription program with digital order entry and prescription printing

Performance tracking	Manual observation; post-hoc documentation	Automated logging of completion times, task sequences, and departmental handoff timestamps
Evaluation method	Subjective observer assessment	Operational readiness checklist with automated metrics from platform logs
Repeatability	Limited to 1–2 sessions/year; high resource requirements per session	Scenarios can be reset and re-executed without additional physical setup

Quantitative results (training duration, FTE values, completion rates) are reported in the Results section of the main manuscript.

Qualitative Data Collection and Analysis

Following the training session and debriefing (July 3, 2024), qualitative feedback was collected from all 60 participants through open-ended written responses (**Supplementary Table S7**). Participants provided their overall impressions, platform improvement suggestions, and ideas for future training scenarios. Responses were organized by the 7 functional groups.

Supplementary Table S7. Open-ended survey instrument administered to participants

Field	Description
Survey item	"Please describe freely your thoughts on the digital twin-based IT outage disaster response simulation training (e.g., opinions on the training, suggestions for future training)."
Item type	Single open-ended free-text item
Administration	Anonymous, paper-based, self-administered, distributed and collected immediately following the training session on July 3, 2024
Respondents	All 60 multidisciplinary training participants who had provided prior consent (response rate 100%)
Consent statement	"This survey is conducted to support the improvement of the digital twin-based virtual hospital platform and the future development of disaster response systems. Responses will be processed anonymously and may be used for research purposes including publications and academic dissemination. Do you consent to your anonymized responses being used for research purposes?" (Yes / No)
Analysis	Structured content summarization by two independent researchers, with text mining and word cloud analysis applied in parallel. Five content categories were derived inductively (see Table 4 in the main manuscript).

The analysis followed a structured content summarization approach. Two researchers (S.A.B. and Y.J.K.) independently read all responses and generated initial descriptive codes. Codes were organized in a spreadsheet and grouped into similar categories through discussion. Five thematic categories were derived: target extensions, mock training ideas, drug/prescription system improvement, operations and evaluation systems, and process improvement. Text mining and word cloud analysis were applied in parallel to identify the most frequently mentioned keywords.

The qualitative component was designed as a supplementary evaluation to capture participant perspectives and generate actionable improvement suggestions, rather than as a standalone qualitative research study.

Pre-Training Education Program

Two structured education sessions were conducted prior to the exercise. Session 1 (June 27, 2024) was a 2-hour session attended by 30 participants. Content included: (a) digital twin platform access and navigation practice using the dashboard, (b) emergency prescription program instructional video, and (c) hands-on practice covering patient registration, order entry, order verification, specimen management, and medication dispensing workflows using a practice scenario.

Session 2 (July 2, 2024) was a focused session for 5 Medical Information Technology team members who served as training facilitators. Content included verification of registered master data and hands-on practice of training operation and monitoring procedures. The overall training execution workflow is illustrated in **Supplementary Figure S2**.

Supplementary Figure S2. Training execution workflow.

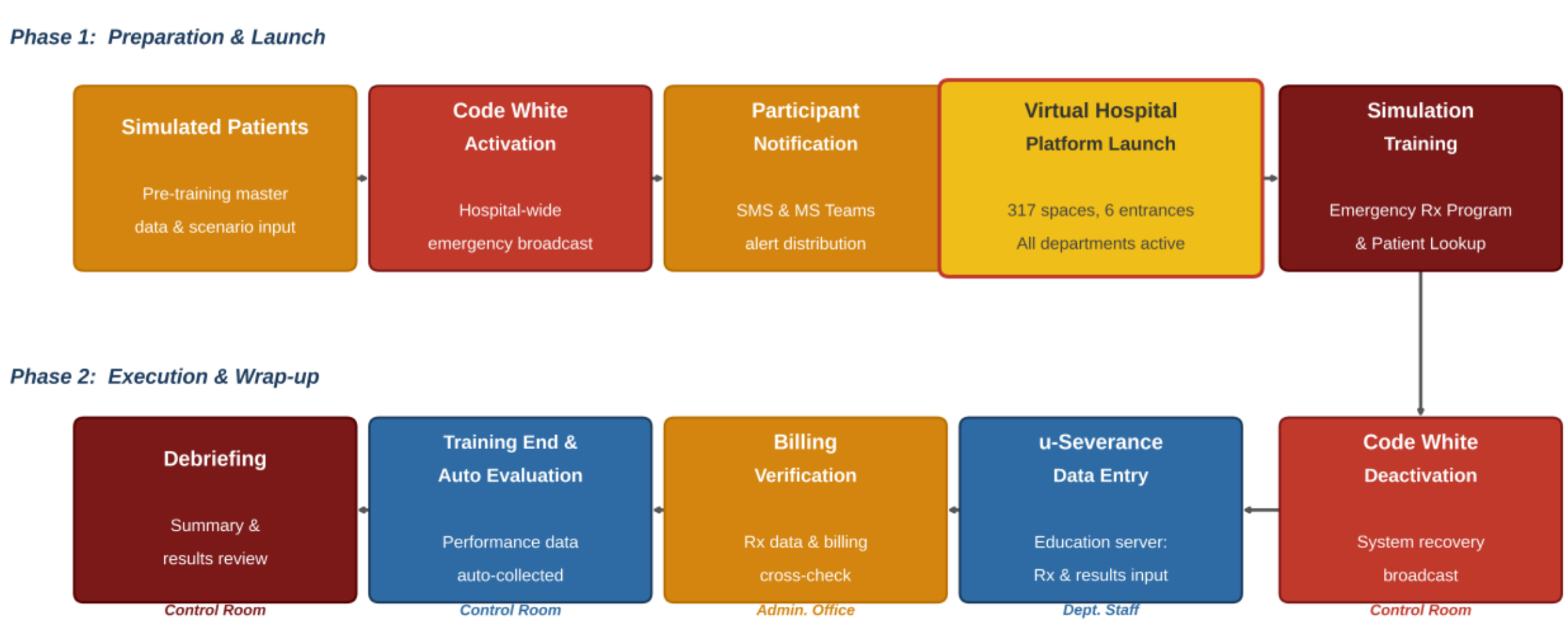

The training execution workflow proceeded in two phases. Phase 1 (Preparation & Launch) covered simulated patient master data input, Code White activation via hospital-wide broadcast, participant notification through SMS and MS Teams, virtual hospital platform launch (317 clinical spaces and 6 entrances), and initiation of simulation training using the emergency prescription program and patient lookup system. Phase 2 (Execution & Wrap-up) covered Code White deactivation upon simulated system recovery, retrospective data entry into u-Severance (education server), prescription and billing cross-verification by the Administrative Office, automated performance evaluation data collection, and debriefing.

Emergency Prescription Program

The MS Teams-based emergency prescription program was developed in-house over approximately 5 months (July–December 2023). It was designed as a functional replacement for the hospital's order communication system (u-Severance) during IT outage, supporting: (a) patient registration and lookup, including new patient creation and automated billing status verification; (b) order entry for laboratory tests, imaging studies, and pharmacy orders; (c) real-time order status tracking across departments; (d) post-payment confirmation and receipt generation; and (e) prescription printing with labels indicating "IT outage emergency prescription." The program underwent 23 documented development iterations, including migration of hospital master databases and integration of 178 laboratory test items (of 2,122 total) feasible during outage conditions.
